# Understanding of chiral site-dependent enantioselective identification on a plasmon-free semiconductor based SERS substrate†

**DOI:** 10.1039/d2sc01938h

**Published:** 2022-05-11

**Authors:** Jing Xu, Yuanfei Xue, Xiaoxia Jian, Yue Zhao, Zhenqing Dai, Jingwen Xu, Zhida Gao, Ye Mei, Yan-Yan Song

**Affiliations:** College of Sciences, Northeastern University Shenyang 110819 China yysong@mail.neu.edu.cn; State Key Laboratory of Precision Spectroscopy, School of Physics and Electronic Science, East China Normal University Shanghai 200062 China ymei@phy.ecnu.edu.cn; NYU-ECNU Center for Computational Chemistry at NYU Shanghai Shanghai 200062 China; Collaborative Innovation Center of Extreme Optics, Shanxi University Taiyuan Shanxi 030006 China

## Abstract

Chiral differentiation is an important topic in diverse fields ranging from pharmaceutics to chiral synthesis. The improvement of sensitivity and the elucidation of the mechanism of chiral recognition are still the two main challenges. Herein, a plasmon-free semiconductive surface-enhanced Raman spectroscopy (SERS) substrate with sensitive chiral recognition ability is proposed for the discrimination of enantiomers. A homochiral environment is constructed by typical π–π stacking between l-tryptophan (l-Trp) and phenyl rings on well-aligned TiO_2_ nanotubes (TiO_2_ NTs). Using 3,4-dihydroxyphenylalanine (DOPA) enantiomers as the targets and the chelating interaction of Fe^3+^–DOPA for the onsite growth of Prussian blue (PB), the enantioselectivity difference between l-DOPA and d-DOPA on the homochiral substrate can be directly monitored from PB signals in the Raman-silent region. By combining the experimental results with molecular dynamic (MD) simulations, it is found that satisfactory enantioselective identification not only requires a homochiral surface but also largely depends on the chiral center environment-differentiated hydrogen-bond formation availability.

## Abstract 2

An intelligent enantioselective identification strategy is designed to demonstrate that both enantioselectivity and stereoselectivity are crucial factors for chiral sensing.

## Graphical abstract

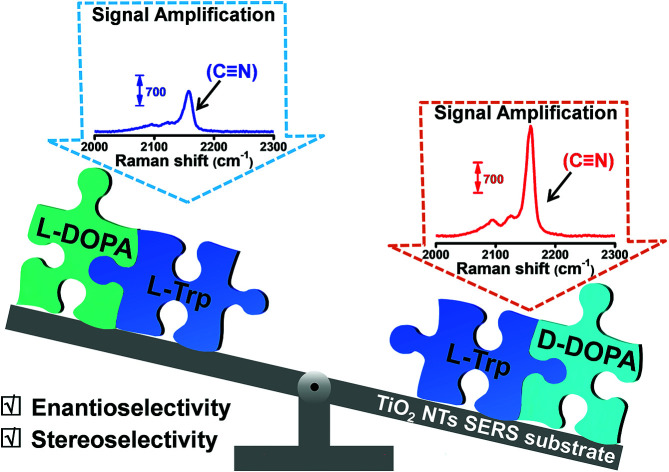

## Introduction

Chirality, one of the most important characteristics of chiral materials, plays an important role in life sciences. Most biologically active compounds (amino acids, sugars, peptides, proteins, *etc.*) and modern drugs are chiral.^1–4^ Although enantiomers have similar physicochemical properties, they exhibit completely different physiological effects in terms of biological activity, toxicity, and pharmacological actions.^5^ A characteristic example of enantiomers is 3,4-dihydroxyphenylalanine (DOPA). l-DOPA is a common drug used for the treatment of Parkinson's disease. However, the d-enantiomer exhibits neurotoxicity.^6^ Therefore, the identification and quantification of enantiomeric forms of chiral molecules are important in chemistry, biology, and pharmaceutical sciences. In the past decade, much effort has been devoted to the study of enantiomeric discrimination, and a variety of chiral sensing techniques have been developed, including vibrational circular dichroism (VCD),^7^ high-performance liquid chromatography (HPLC),^8^ and electrochemical assay.^9^ Although these technologies have helped to make great progress in chiral discrimination, the construction of sensitive and low-cost enantiomer recognition detection platforms and elucidation of the mechanism of enantioselective recognition are still two great challenges in the design and development of highly effective homochiral drugs.

In general, the conventional chiral recognition methods rely on the inherent optical activity of chiral molecules, in which circularly polarized light is a prerequisite for distinguishing two enantiomers.^10,11^ The other types of recognition methods need to choose a chiral molecule as the enantioselector. The identification of an enantiomer is achieved based on the difference in the stereostructure of a chiral selector and each of the enantiomers.^12–15^ The recognition mechanism commonly follows the three-point interaction principle, *i.e.*, only one enantiomer can simultaneously form three interactions with the selector while the other cannot.^16,17^ Therefore, the study of the binding sites of enantiomeric molecules and selectors can help us to better understand the recognition mechanism of chiral molecules and construct an enantioselective platform for the identification of enantiomers.

Surface-enhanced Raman spectroscopy (SERS) is a nondestructive but sensitive analytical technique that features unique molecular structural fingerprint information. In principle, the SERS effect is mainly attributed to two mechanisms: (i) strong electromagnetic mechanism (EM) induced by surface plasmon resonance (SPR) and (ii) chemical mechanism (CM) induced by dipole–dipole interactions or charge-transfer resonances between the SERS substrates and probe molecules.^18–24^ Traditionally, owing to the excellent SPR effect, noble metals (Au, Ag, and Cu) have been widely used as substrate materials for SERS with a detection limit as low as 10^−10^ M. On the other hand, because of a large number of structural defects, modulated surface sites, and layer-number-dependent bandgap, two-dimensional (2D) semiconductor nanosheets (TiO_2_, MoS_2_, ZnO, and WO_3_) have been discovered as alternatives to noble-metal-based SERS substrates.^25–29^ Nevertheless, besides the poor stability of ultrathin materials, the reproducibility of preparing a uniform distribution on carrier surfaces is another challenge for the practical applications of these nanosheet-like semiconductors in SERS.

**Fig. 1:**
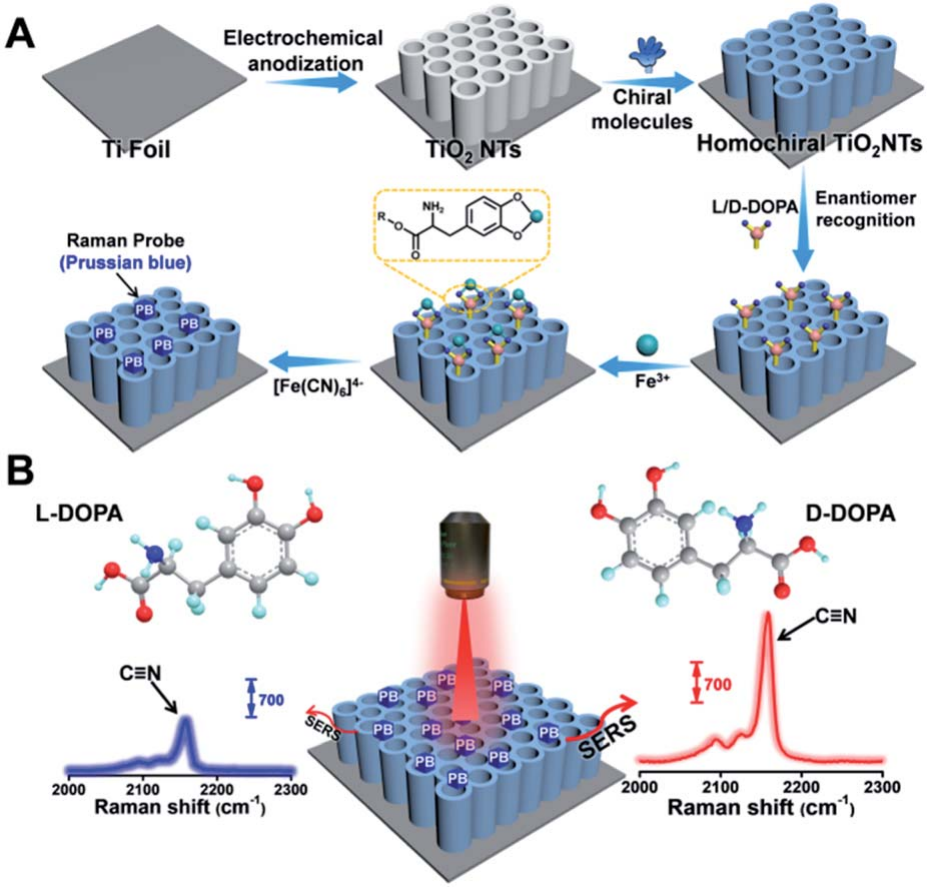
(A) Fabrication of a homochiral SERS substrate and PB generation. (B) SERS-based enantiomeric recognition and qualification of DOPA enantiomers *via* the PB signal at 2158 cm^−1^.

Recently, Weidinger' group has confirmed that in addition to sheet-like semiconductor materials, TiO_2_ nanotubes (TiO_2_ NTs) also exhibit good SERS performance.^30^ The three-dimensional (3D) periodically ordered nanostructures acted as photonic lattices that reflect light of certain frequencies, thus enabling a noticeable enhancement in the Raman signal.^31^ Owing to their unique biocompatibility, surface groups, chemical stability, and surface morphology, TiO_2_ NTs are promising SERS substrates. In this study, utilizing the affinity interaction between phosphonic acid (PA) and the TiO_2_ surface, as well as the well-known π–π stacking interaction between the phenyl rings on TiO_2_ NTs and l-tryptophan (l-Trp), we developed a simple but effective strategy to construct homochiral SERS substrates for enantioselective recognition. Considering a proof-of-concept application, l/d-DOPA was selected as the analyte of interest, and a background-free Raman reporter Prussian blue (PB) was generated onsite by reacting Fe^3+^–DOPA chelated complexes with [Fe(CN)_6_]^4−^ (Fig. 1A). On the basis of the nitrile vibration peak of PB at 2158 cm^−1^ in the Raman-silent region,^32^ the enantioselective recognition between l/d-DOPA and the homochiral SERS substrate can be easily determined from the peak intensity (Fig. 1B). By combining the Raman results with quantum mechanical calculation, the chiral center-environment-differentiated hydrogen bond formation availability was simulated to better understand the recognition mechanism. Furthermore, to confirm the role of the chiral center environment, a homochiral environment based on direct amide bonding between the chiral center and SERS substrate was also constructed and demonstrated with a negligible difference in chiral discrimination. This study provides an intelligent and sensitive strategy for the selective recognition of chiral molecules on a noble-metal-free SERS substrate.

## Results and discussion

### Preparation and characterization of the plasmon-free SERS substrate

**Fig. 2:**
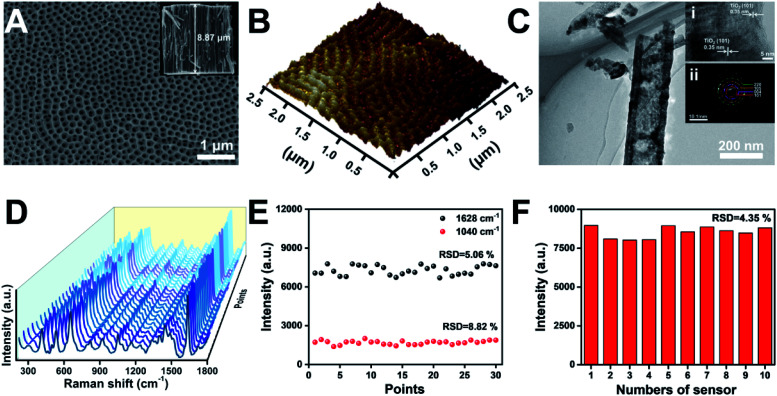
(A) SEM images of the top view and cross-sectional view (inset) of TiO_2_ NTs. (B) AFM image of TiO_2_ NTs. (C) TEM, high-resolution transmission electron microscopy (HR-TEM, inset (i)), and SAED (inset (ii)) images of TiO_2_ NTs. (D) SERS spectra of MB molecules (2 × 10^−5^ M) collected on the thirty random points of TiO_2_ NTs, and (E) the corresponding signal intensity at 1040 cm^−1^ and 1628 cm^−1^. (F) Signal intensity of MB at 1628 cm^−1^ collected on ten TiO_2_ NT based substrates to convey reproducibility.

The application of TiO_2_ NTs as a SERS substrate for chiral molecule detection is shown in Fig. 1. TiO_2_ NTs were grown by the electrochemical anodization of Ti foil.^33^ As shown in the scanning electron microscopy (SEM) images, the well-aligned TiO_2_ NTs directly grown on a Ti substrate are composed of numerous nanotubes with good uniformity and a tube diameter of ∼115 nm (Fig. 2A). The self-standing NTs are scalable and have good repeatability, thus solving the challenges when using semiconductor nanosheets as SERS substrates. The atomic force microscopy (AFM) image also confirms that the TiO_2_ NTs are highly ordered with a uniform distribution of pores over a long range (Fig. 2B). As further characterized by transmission electron microscopy (TEM), the tube wall of TiO_2_ NTs is ∼41 nm, and a lattice spacing of 0.35 nm can be ascribed to the (101) plane of the anatase TiO_2_ structure (Fig. 2C). The as-formed TiO_2_ NTs have a higher crystallinity as indexed in the selected-area electron diffraction (SAED) image.

Generally, in addition to the chemical enhancement effect caused by the formation of charge-transfer complexes between the adsorbate and surface, the enhanced localized electric fields resulting from the specific optical properties of the nanotubular geometry are identified as the dominant factor for Raman signal amplification.^30,31^ To achieve a satisfactory Raman signal on TiO_2_ NT based SERS substrates, the tube length was optimized using methylene blue (MB) as a Raman probe. As the tube length is mainly determined by the anodization time (Fig. S1†), the SERS performance of TiO_2_ NTs prepared at different anodization time periods was studied (Fig. S2†). Compared with TiO_2_ nanoparticle based membranes, the NTs show stronger SERS signals. Owing to the periodic geometric morphology of TiO_2_ NTs, multiple laser scattering among the periodic voids improved the light–matter interaction and provided much more opportunities for the occurrence of Raman scattering. Furthermore, the enrichment effect of the nanotubular geometry structure with a higher specific surface area can also be attributed to the increased SERS signal. The SERS signals increase with the length of TiO_2_ NTs. However, with further increase of the tube length, the SERS signals start to decrease. This is because a part of the scattered light was trapped in the nanotubes, resulting in scattering loss.^34^ In the following study, ∼8.87 μm TiO_2_ NTs were used as the SERS substrate (inset in Fig. 2A). The enhancement factor (EF) of TiO_2_ NTs was determined to be 2.7 × 10^4^ based on the MB SERS signal on TiO_2_ NTs and a silicon wafer (Fig. S3†). The signal uniformity was investigated by the acquisition of Raman spectra at thirty random points (Fig. 2D). As plotted in Fig. 2E, the signal intensity of the Raman peak at 1040 and 1628 cm^−1^ exhibits satisfactory uniformity. Furthermore, the reproducibility was also investigated by comparing the Raman signals on ten TiO_2_ NT samples that were prepared using the same process (Fig. S4†). As shown in Fig. 2F, the relative standard difference (RSD) for these samples is only 4.35%. These results show good reliability and application potential of the TiO_2_ NT based SERS substrate in molecular sensing.

### Enantioselective recognition and Raman sensing

**Fig. 3:**
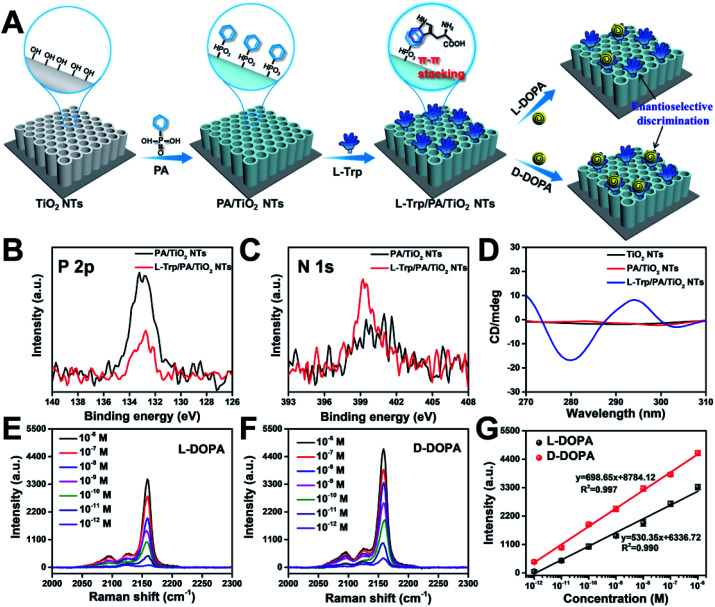
(A) Schematic illustrations of constructing a homochiral environment by π–π stacking for sensing DOPA enantiomers. XPS signals of (B) P 2p and (C) N 1s before and after l-Trp was anchored onto PA/TiO_2_ NTs. (D) CD spectra of TiO_2_ NTs, PA/TiO_2_ NTs, and l-Trp/PA/TiO_2_ NTs. SERS spectra of PB for sensing different concentrations of (E) l-DOPA and (F) d-DOPA on l-Trp/PA/TiO_2_ NTs, and (G) the corresponding Raman intensities at 2158 cm^−1^.

To achieve a satisfactory sensitivity and selectivity in enantioselective identification, the homochiral environment and signal amplification are the two crucial issues. Therefore, TiO_2_ NT based chiral recognition systems contain two parts: enantioselective identification and signal generation (Fig. 1A). As a proof-of-concept for enantiomer recognition, DOPA enantiomers were used as the targets to understand the chiral sensing ability of the as-proposed SERS substrate and elucidate the mechanism in enantioselective identification. Fig. 3A shows the step-by-step modification details for constructing a homochiral environment and l/d-DOPA recognition. The aromatic amino acid l-Trp was used as the chiral host in this study to prepare homochiral substrates. Based on plenty of surface hydroxyl groups (Ti–OH) on the as-prepared TiO_2_ NTs, the PA monolayer can be easily self-assembled on a TiO_2_ surface *via* the well-known affinity interaction between PA and Ti–OH.^35^ In the next step, l-Trp was anchored onto the tube wall by the π–π stacking interactions of the phenyl rings of l-Trp and PA, thus constructing the homochiral environment. X-ray photoelectron spectra (XPS) showed that the resulting sample (l-Trp/PA/TiO_2_ NTs) is composed of Ti, O, P, and N elements without other detectable impurities (Fig. S5†). The appearance of P 2p signals confirms the successful PA anchoring (Fig. 3B). In addition, the characteristic peaks of N 1s at 399.98 eV (Fig. 3C) can be attributed to the amino group and N atom on the pyridine ring, indicating the existence of l-Trp. Furthermore, the energy-dispersive X-ray spectroscopy (EDS) mapping images also showed the well-dispersed P and N elements on the channel wall (Fig. S6†). In the Raman spectrum (Fig. S7†), the characteristic peak at 1000 cm^−1^ (βCCC, benzene ring) further indicates the successful modification of PA.^36^ To identify the homochiral character of the resulting SERS substrate, the circular dichroism (CD) spectra of TiO_2_ NTs after each modification step were obtained (Fig. 3D). An adsorption peak at 294 nm appeared in the CD spectrum of l-Trp/PA/TiO_2_ NTs that can be ascribed to l-Trp (Fig. S8†), indicating the homochirality of the l-Trp/PA/TiO_2_ NT based SERS substrate.

Because of the small Raman cross section of chiral molecules, it is difficult to directly use the characteristic Raman peaks of l/d-DOPA for the sensitive recognition difference (Fig. S9†). To solve this problem, PB, a Raman-silent region probe, was generated onsite *via* the reaction induced by DOPA recognition. As shown in Fig. 1A, the well-known chelation interaction between Fe^3+^ and phenolic hydroxyl groups on DOPA was first used to introduce Fe^3+^ ions.^37,38^ The formed Fe^3+^–catechol coordination complexes then reacted with [Fe(CN)_6_]^4−^ to grow PB nanocrystals onsite. The C≡N vibration in PB resulted in a strong and sharp characteristic peak at 2158 cm^−1^ (Fig. 1B). Therefore, the enantioselective difference of the homochiral SERS substrate for l/d-DOPA recognition could be achieved by simply comparing the characteristic peak of PB. Considering the self-polymerization of DOPA in a basic solution, the chiral recognition was performed under an acidic aqueous solution (pH 5.5).^39^ To obtain a strong PB signal for the sensitive qualification of DOPA, the reaction conditions such as chiral recognition time (Fig. S10†), Fe^3+^ concentration (Fig. S11†), and chelation time (Fig. S12†) were optimized. In this study, the optimal experimental conditions of chiral recognition time, Fe^3+^ concentration, and Fe^3+^ chelation time used in the subsequent experiments are 60 min, 0.25 mM, and 30 min, respectively. Considering that Fe^3+^ ions would be adsorbed on the negatively charged surfaces (Fig. S13†), we also investigated the PB generation possibility on TiO_2_ NTs, PA/TiO_2_ NTs, and l-Trp/PA/TiO_2_ NTs (Fig. S14†). Apparently, very weak PB signals were detected on these samples, indicating negligible interference from the electrostatically adsorbed Fe^3+^ ions on DOPA sensing.

Fig. 3E and F show the Raman spectra of PB for recognizing different concentrations of l-DOPA and d-DOPA, respectively. The Raman intensity increased with increasing l/d-DOPA concentration. More importantly, the as-proposed sensing system showed good enantioselectivity with stronger Raman signals appearing for d-DOPA. The EDS elemental mapping results indicate the formation of PB by the well-dispersed distribution of Fe and N elements (Fig. S15†). In addition, the X-ray diffraction (XRD) patterns (Fig. S16A†) and UV/visible diffuse reflectance absorbance spectra (Fig. S16B†) also provide evidence for the existence of PB nanoparticles. Fig. 3G shows the corresponding relationship between the l/d-DOPA concentration and the intensity of the Raman peak at 2158 cm^−1^. A good linear relationship in the l/d-DOPA concentration was observed from 10^−6^ to 10^−12^ M. The limit of detection (LOD) based on a signal-to-noise ratio of 3 (S/N = 3) was estimated to be as low as 1.7 × 10^−13^ M for l-DOPA and 1.3 × 10^−13^ M for d-DOPA. More importantly, d-DOPA always induced a higher Raman intensity than l-DOPA at the same sample concentration. The detection sensitivity obtained in this study has obvious advantages over the recently reported colorimetric and electrochemical methods (Table S1†). From the Raman intensity, the enantioselectivity coefficient (defined as: *I*_2158_ (d-DOPA)/*I*_2158_ (l-DOPA) to quantify the chiral distinction) was determined to be 7.6 at 10^−12^ M and 1.4 even at a high concentration of 10^−6^ M. Such a difference in SERS intensity can be explained by the discrepancy in the interaction between l-Trp and l/d-DOPA enantiomers: more d-DOPA molecules were captured on the homochiral l-Trp/PA/TiO_2_ NTs. The difference in the PB generation amount on l-Trp/PA/TiO_2_ NTs can be distinguished by SEM characterization (Fig. S17†). Apparently, the nanotube diameter became smaller after the chiral recognition of d-DOPA followed by PB formation (Fig. S17E and F,† 68.2 nm) than that of l-DOPA (Fig. S17C and D,† 75.8 nm).

### Universality and selectivity investigation

To verify the universality of this SERS-sensing strategy, we attempted to apply π–π stacking for constructing chiral environments using other chiral aromatic molecules. For this purpose, l-phenylalanine (l-Phe) was used to replace l-Trp. The adsorption peak at 221 nm in the CD spectrum of l-Phe/PA/TiO_2_ NTs confirmed the homochiral properties of the resulting SERS substrate (Fig. S18†). Under the optimized conditions, l-Phe/PA/TiO_2_ NTs showed a remarkable enantioselectivity for l/d-DOPA with a distinct SERS signal of PB (Fig. S19†). The selectivity of the chiral sensing system was also evaluated by the identification of other enantiomers, involving l/d-Phe, l/d-alanine (l/d-Ala), l/d-proline (l/d-Pro), l/d-glutamic acid (l/d-Glu), and l/d-histidine (l/d-His) (Fig. S20†). The obvious PB signal at 2158 cm^−1^ was only observed for l/d-DOPA recognition (Fig. S21†), which can be ascribed to the special chelation interaction between Fe^3+^ and phenolic hydroxyl groups on DOPA. These results indicate that the as-proposed SERS-based chiral sensing system obtained using chiral aromatic molecules to construct homochiral environments has satisfactory enantioselectivity, versatility, and reliability.

### Mechanism of enantioselective identification

**Fig. 4:**
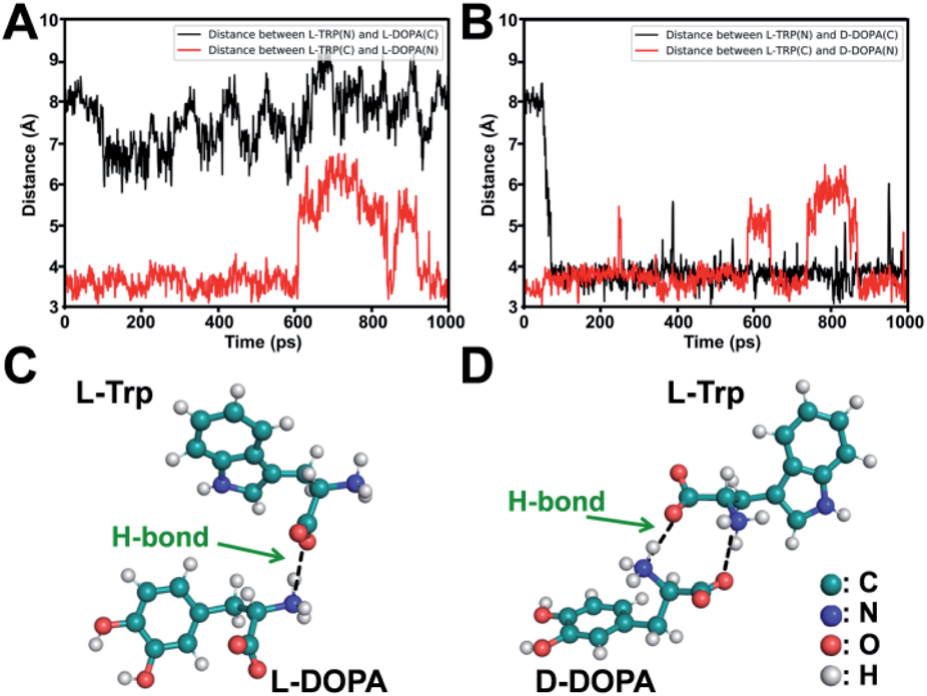
(A) Distance between the –COO^−^ and –NH_3_^+^ groups of l-Trp and l-DOPA. (B) Distance between the –COO^−^ and –NH_3_^+^ groups of l-Trp and d-DOPA. Energy-minimized dominant interaction models of (C) l-Trp with l-DOPA and (D) l-Trp with d-DOPA, shown as a ball-and-stick model.

To elucidate the underlying mechanism, we conducted molecular dynamics (MD) simulations for the recognition of DOPA enantiomers on the chiral site of l-Trp. It was suggested that the hydrogen bond formed by–NH_3_^+^/–COO^−^ interaction would be the strongest interaction in this chiral recognition system.^40^ The distance between –NH_3_^+^ and –COO^−^ was monitored to determine the chiral recognition difference of the enantiomers on the homochiral l-Trp/PA/TiO_2_ NTs, which serves as an indicator for the stability of H bonds. We calculated the distances between (i) the –NH_3_^+^ group on the chiral site for l-Trp (denoted as l-Trp (N)) and the –COO^−^ group of DOPA (denoted as DOPA (C)); (ii) the –COO^−^ groups of l-Trp (denoted as l-Trp (C)) and –NH_3_^+^ of DOPA (denoted as DOPA (N)). For l-DOPA, as shown in Fig. 4A, the distance between l-Trp (N) and DOPA (C) is larger than that between l-Trp (C) and DOPA (N) (within 4 Å) during the simulation, suggesting that the –NH_3_^+^ groups of l-DOPA are available for binding onto the homochiral substrate *via* the formation of H bonds compared with the –COO^−^ groups of l-DOPA. Fig. 4B shows that the distance between l-Trp (N) and d-DOPA (C) as well as the distance between l-Trp (C) and d-DOPA (N) are both in a short range (within 4 Å). These results indicate that two types of H bonds are favorable for d-DOPA recognition. Because of the energetically favorable stereoselectivity, the –NH_3_^+^ and –COO^−^ groups in both molecules are paired with each other. As shown in Fig. 4C, only one H bond is expected to be formed between l-Trp and l-DOPA. For comparison, two H bonds would be formed between l-Trp and d-DOPA (Fig. 4D). In this case, the two H bonds largely strengthen the interactions between l-Trp and d-DOPA and thus more d-DOPA is recognized on the homochiral l-Trp/PA/TiO_2_ NTs. As a result, a larger number of Fe^3+^ ions were anchored on the substrate in the subsequent step, and through the PB generation step, the enantioselective identification can be directly determined from the Raman signal of PB.

**Fig. 5:**
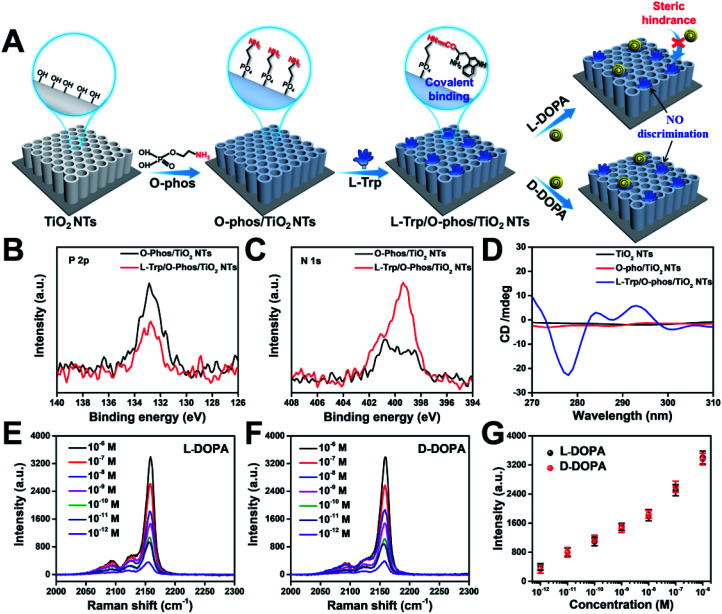
(A) Schematic illustrations of constructing a homochiral environment by covalent amide bond formation for sensing DOPA enantiomers. XPS signals of (B) P 2p and (C) N 1s before and after l-Trp was anchored onto *O*-phos/TiO_2_ NTs. (D) CD spectra of TiO_2_ NTs, *O*-phos/TiO_2_ NTs, and l-Trp/*O*-phos/TiO_2_ NTs. SERS spectra of PB for sensing different concentrations of (E) l-DOPA and (F) d-DOPA on l-Trp/*O*-phos/TiO_2_ NTs, and (G) the corresponding Raman intensities at 2158 cm^−1^.

Stereoselectivity is also crucial for chiral binding sites in the enantiomer recognition event. To demonstrate the key role of stereoselectivity, l-Trp was linked with TiO_2_ NTs *via* an amide bond by utilizing the –COOH group on the chiral carbon of l-Trp and –NH_2_ groups on the TiO_2_ surface. As shown in Fig. 5A, the –NH_2_ group was first modified on TiO_2_ NTs by the condensation reaction between P–OH groups in *o*-phosphorylethanolamine (*O*-phos) and Ti–OH groups.^35^ The grafting of the *O*-phos monolayer was confirmed by XPS analysis from the appearance of P 2p signals (Fig. 5B). Then, l-Trp was introduced by forming an amide bond through a classic *N*-(3-dimethylaminopropyl)-*N*-ethylcarbodiimide hydrochloride (EDC)/*N*-hydroxysulfosuccinimide (NHS) coupling reaction,^41^ in which the –COOH group on a chiral site is used for forming an amide bond with –NH_2_ groups on the tube wall. The successful covering of l-Trp on *O*-phos/TiO_2_ NTs leads to the decrease of P 2p signals (Fig. 5B) as well as the increase of N 1s signals (Fig. 5C). The homochiral feature was confirmed from CD spectra with the appearance of an adsorption peak at 294 nm (Fig. 5D). Notably, the PB signals obtained from l-DOPA and d-DOPA recognition on the l-Trp/*O*-phos/TiO_2_ NT substrate show almost the same intensities (Fig. 5E–G), indicating that the homochiral substrate has a similar recognition ability for the targeted enantiomers. Such a phenomenon can be attributed to the steric hindrance from the indole group on l-Trp. Because of the steric hindrance from the indole group, DOPA recognition is originated from the H bonds formed between the –NH– on pyrrole and l/d-DOPA. As it is difficult to reach the chiral site of l-Trp, DOPA enantiomers exhibit the same recognition ability on l-Trp/*O*-phos/TiO_2_ NTs. These results indicate that even though the substrate is homochiral, stereoselectivity, especially the steric hindrance at a chiral site, is an important factor that should be considered for designing an enantioselective material.

## Experimental

### Preparation of TiO_2_ NTs and TiO_2_ nanoparticle based membrane

The TiO_2_ NTs were grown on Ti substrates (15 mm × 15 mm × 0.1 mm) by electrochemical anodization. The Ti substrates were first ultrasonically cleaned in isopropanol, ethanol, and deionized water in sequence and dried under a N_2_ gas flow. Anodization was carried out in an ethylene glycol/lactic acid/water/NH_4_F electrolyte containing 0.1 M NH_4_F at 120 V for 4 min. Ti substrates and platinum foil served as the working electrode and counter electrode, respectively. A compact TiO_2_ nanoparticle based membrane was grown by the anodization of the Ti substrate in 1 M H_2_SO_4_ at 20 V for 20 min. The prepared samples were annealed at 450 °C for 2 h in air at a heating rate of 3 °C min^−1^.

### Synthesis of l-Trp/PA/TiO_2_ NTs

The as-formed TiO_2_ NTs were first functionalized with PA by immersing the sample in a 10 mM PA ethanol solution at 4 °C for 12 h, followed by rinsing with ethanol three times. Then, chiral recognition molecules (l-Trp) were introduced onto the prepared PA/TiO_2_ NTs through π–π stacking interaction. The substrates were soaked in a 1 mM l-Trp aqueous solution at room temperature for 12 h, producing l-Trp/PA/TiO_2_ NTs.

### Detection of l/d-DOPA

The as-prepared l-Trp/PA/TiO_2_ NTs were first immersed in an aqueous solution of l/d-DOPA (pH 5.5) at room temperature for 60 min and then lightly cleaned with deionized water three times and dried under a N_2_ stream. These samples were then incubated in FeCl_3_ aqueous solution for 30 min to anchor Fe^3+^ ions, and then gently washed with deionized water to remove the electrostatically adsorbed Fe^3+^ ions. After that, PB was generated by soaking the samples in a 0.25 mM K_4_[Fe(CN)_6_] aqueous solution at room temperature for 30 min. The resultant samples were washed with deionized water three times and dried with N_2_ gas.

### Raman measurement

MB ethanol solutions were used as the analyte. Then, 200 μL of MB solution was added dropwise and spread on a substrate (50 mm × 50 mm scale) and dried in the dark. Raman spectra were recorded using a 638 nm laser (10% power) equipped with a 50× long-distance objective lens and a 1 μm spot size. The data acquisition time was kept as 10 s; the confocal hole size was 500 μm; the slit aperture size was 100 μm. The spectrometer was calibrated using the Raman spectra of silicon wafer at 520.7 cm^−1^. Raman spectra were collected from five different places, and then the average signal intensity was determined.

### EF measurement

As a SERS sample, 4 μL of 2 × 10^−5^ M MB solution was added dropwise and spread on the TiO_2_ NT substrate (50 mm × 50 mm scale) and dried at room temperature. As a non-SERS sample, 20 μL of 2 × 10^−3^ M MB ethanol solution was added dropwise and spread on a bare silicon wafer (50 mm × 50 mm scale) and dried at room temperature. The Raman spectra were recorded using a 638 nm wavelength laser (10% power) with a 50× objective, and the data acquisition time was 10 s.

### MD simulations

The initial structures of l-Trp and DOPA were constructed using GaussView 6 (ref. 42) and optimized at the B3LYP/6-31G(d) level of theory using the Gaussian 16 package.^43^ Possible binding modes between l-Trp and l/d-DOPA were predicted using AutoDock Vina 1.1.2.^44^ For each dimer, nine docked conformations were selected for the simulations. Trajectories containing the following two conditions were selected for subsequent analysis: (1) the initial structure contained at least one NH_3_^+^–COO^−^ interaction, and (2) the dimer did not dissociate in 1 ns simulations. A general AMBER force field (GAFF)^45^ was applied to the molecules, and the AM1 bcc charges were calculated with the optimized structures of l-Trp and l/d-DOPA. l-Trp and l/d-DOPA were dissolved in a periodic cubic TIP3P^46^ water box with the distance between the solute and the box boundary no less than 22.5 Å. All the input files containing parameters and configurations were generated with the LEaP module in AmberTools 19.^47^ The whole systems were optimized for 1000 steps and then heated up to 298.15 K in 100 ps using Langevin dynamics^48^ with a collision frequency of 1 ps^−1^. The nonbonded cutoff was set to 8 Å. The pressure was regulated at 1 atm using Berendsen's scheme. Configurations were collected at 298.15 K with a 1 ps interval from each 1 ns simulation. All the simulations were conducted using AMBER 18.^47^

## Conclusions

In summary, we constructed a chiral sensing platform with a high enantioselectivity on a plasmon-free TiO_2_ NT based SERS substrate. The homochiral environment was constructed by utilizing typical π–π stacking between l-Trp and PA-modified TiO_2_ NTs. The formed l-Trp/PA/TiO_2_ NTs exhibited very good stereoselectivity for d-DOPA over l-DOPA. Benefiting from the chelation binding between DOPA and Fe^3+^ ions, a simple signal output strategy was thus achieved by forming a Raman reporter PB onsite *via* the reaction of Fe^3+^–DOPA and [Fe(CN)_6_]^4−^, providing a sensitive and selective approach for l/d-DOPA recognition and quantification. The mechanism for identification of the difference on the homochiral substrate was well studied by conducting MD simulations and experiments; the results indicate that both enantioselectivity and stereoselectivity are crucial factors for chiral sensing.

## Data availability

The data supporting the findings of this study are available within the article and in the ESI.†

## Author contributions

Y.-Y. Song and Y. Mei conceived the concept and directed the project. J. Xu, X. X. Jian, Y. Zhao, and Z. Q. Dai performed the experiments. Y. F. Xue carried out the theoretical study. J. W. Xu and Z. D. Gao collected and analyzed the data. J. Xu prepared the first draft of this manuscript, and all the authors modified the manuscript.

## Conflicts of interest

There are no conflicts to declare.

## Supplementary Material

SC-013-D2SC01938H-s001
