# Supplementary material for: Understanding of chiral site-dependent enantioselective identification on a plasmon-free semiconductor based SERS substrate
Source: Chem Sci. 2022 May 11;13(22):6550–7. doi: 10.1039/d2sc01938h (PMC9172570; doi:10.1039/d2sc01938h)
Supplement: SC-013-D2SC01938H-s001 [file SC-013-D2SC01938H-s001.pdf]

Electronic Supplementary Material (ESI) for Chemical Science.
This journal is © The Royal Society of Chemistry 2022

Supporting Information

Understanding of chiral site-dependent enantioselective identification on plasmon-free semiconductor based SERS substrate

Jing Xu,[a] Yuanfei Xue,[b] Xiaoxia Jian,[a] Yue Zhao,[a] Zhenqing Dai,[a] Jingwen Xu,[a] Zhida Gao,[a] Ye Mei*[b] and Yan-Yan Song*[a]

[a]College of Sciences, Northeastern University, Shenyang 110819, China

[b]State Key Laboratory of Precision Spectroscopy, School of Physics and Electronic Science, East China Normal University, Shanghai 200062, China; NYU-ECNU Center for Computational Chemistry at NYU Shanghai, Shanghai 200062, China; Collaborative Innovation Center of Extreme Optics, Shanxi University, Taiyuan, Shanxi 030006, China

Corresponding Authors

* Yan-Yan Song, E-mail: yysong@mail.neu.edu.cn.

* Ye Mei, E-mail: ymei@phy.ecnu.edu.cn.

EXPERIMENTAL SECTION

Materials and Reagents. Ti sheets (~0.1 mm thickness, 99.6% purity) were purchased from Baosheng Hardware (Baoji, China). Phenylphosphonic acid ($C_6H_7O_3P$), o-phosphorylethanolamine ($C_2H_8NO_4P$), tryptophan ($C_{11}H_{12}N_2O_2$), phenylalanine ($C_9H_{11}NO_2$), 3,4-Dihydroxy-L/D-phenylalanine ($C_9H_{11}NO_4$), histidine ($C_6H_9N_3O_2$), potassium ferrocyanide trihydrate ($K_4FeC_6N_6 \cdot 3H_2O$), and iron chloride hexahydrate ($FeCl_3 \cdot 6H_2O$) were purchased from Aladdin (Shanghai, China). N-Hydroxysuccinimide (NHS) and N-(3-Dimethylaminopropyl)-3-ethylcarbodiimide hydrochloride (EDC) were obtained from Sigma-Aldrich (St. Louis, USA). Other reagents including ammonium fluoride (NH_4F), isopropyl alcohol, ethylene glycol, and lactic acid were purchased from Sinopharm Chemical Reagent Co. Ltd. and used as received without further purification.

Apparatus and Characterization. Morphological and microstructures characterization were carried out using a field-emission scanning electron microscope (SEM, Hitachi SU8000, Japan). SERS measurements were conducted using a Raman microscopy spectrometer (LabRAM HR, HORIBA Scientific, France). XRD patterns were acquired using an X'Pert XRD spectrometer (Philips, USA) using a CuKα X-ray source. X-ray photoelectron spectra (XPS) were recorded on a Perkin-Elmer Physical Electronics 5600 spectrometer using AlKα radiation at 13 kV as excitation source. UV-visible diffuse reflectance spectra were obtained using a Perkin-Elmer spectrometer (Lambda 750S, USA). Ultraviolet visible (UV/Vis) diffuse reflectance spectra were recorded using a Lambda Bio/XLS+ UV-vis spectrophotometer (PerkinElmer, USA) with a 1.0 cm optical path.

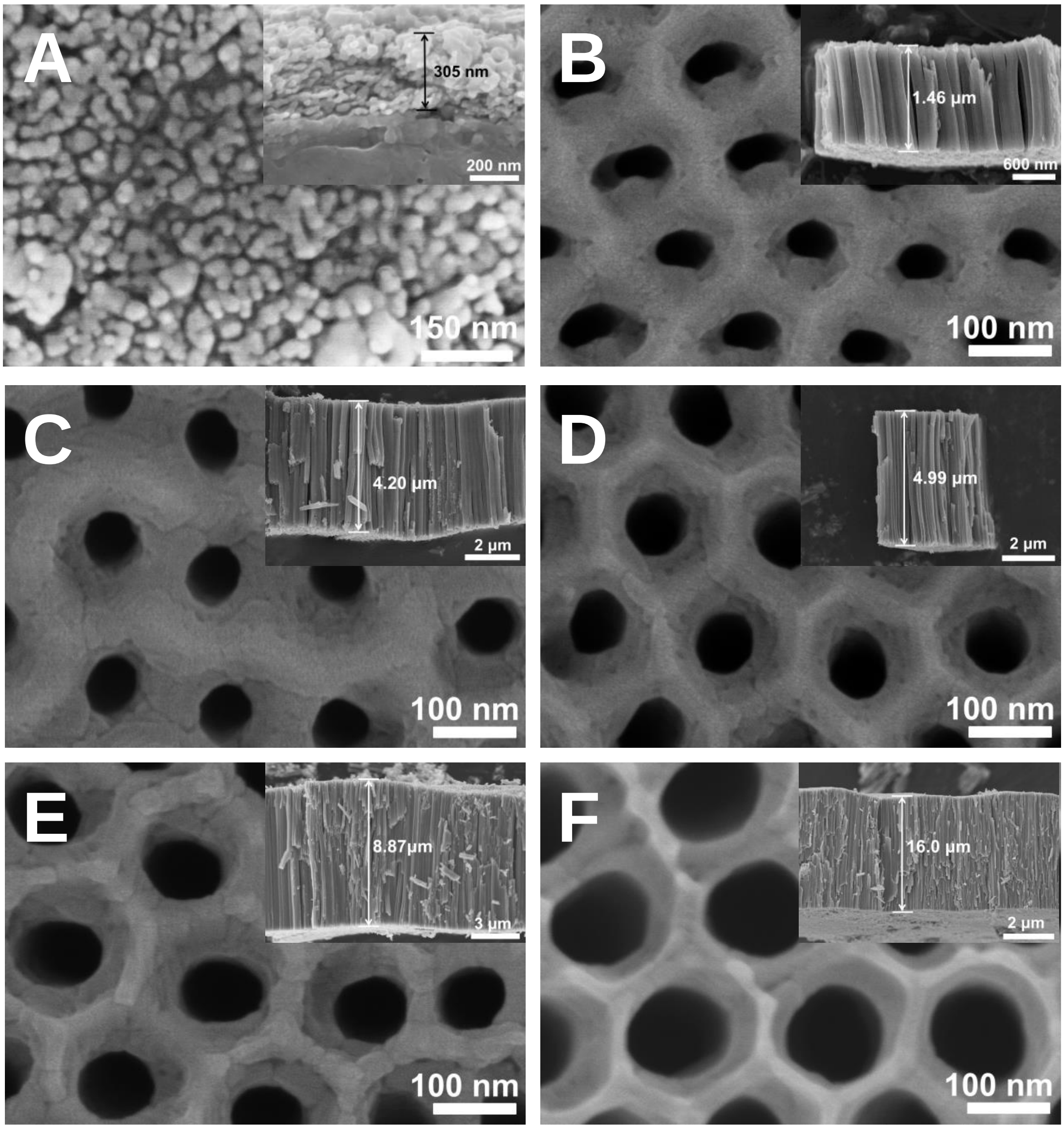

Fig. S1. SEM images of top view and side view of (A) TiO_2 nanoparticles based membrane, and TiO_2 NTs prepared with different anodization periods: (B) 1 min, (C) 2 min, (D) 3 min, (E) 4 min, and (F) 10 min.

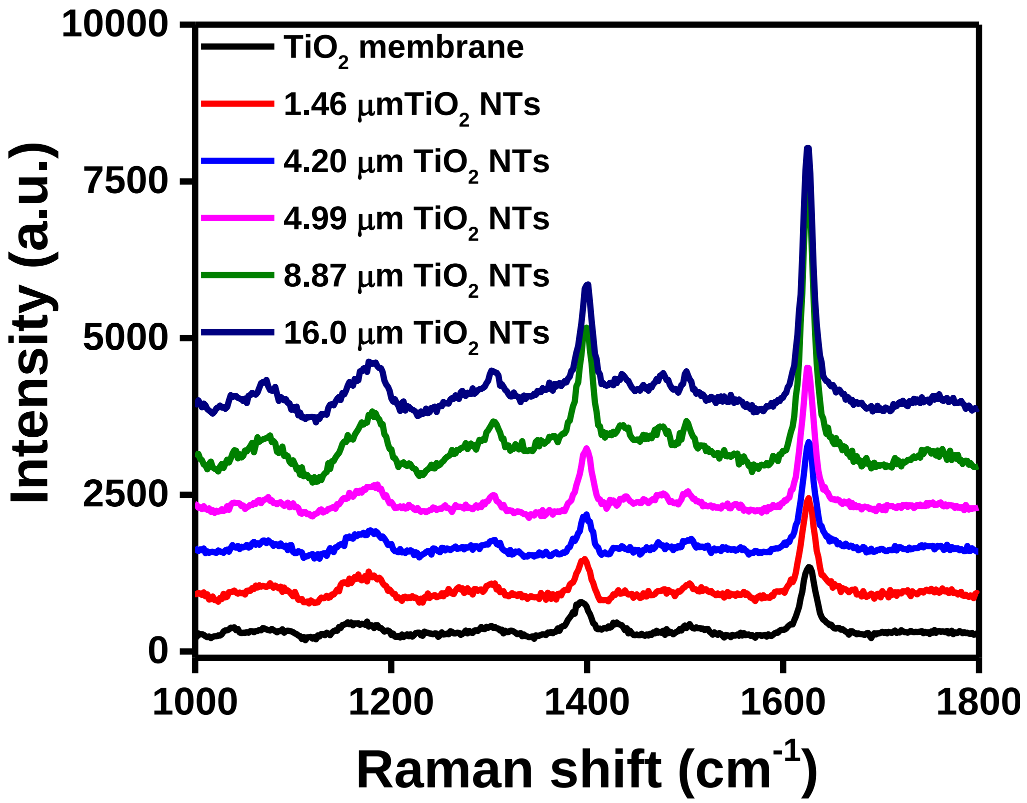

Fig. S2. SERS spectra of MB adsorbed on TiO_2 NTs with different lengths.

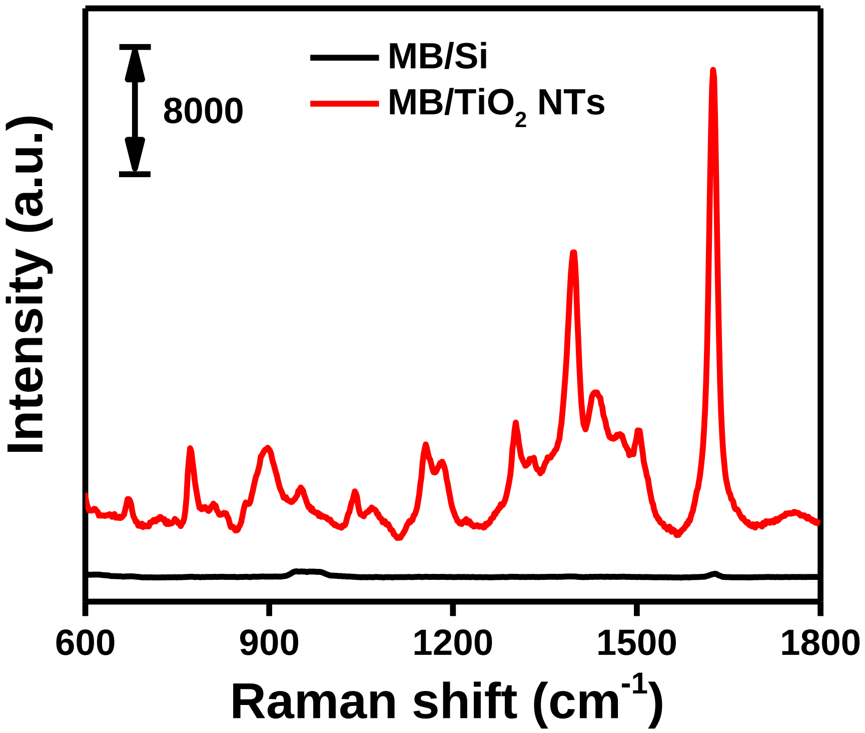

Fig. S3. Raman spectra of MB adsorbed on different substrates: silicon wafer and TiO_2 NTs.

The enhancement factor (EF) of TiO_2 NTs was evaluated using Eq 1.[S1] I_{SERS} and I_{bulk} are the intensities of the 1628 cm^{-1} (C-C stretching vibration) band of MB adsorbed on a SERS-active substrate and bare silicon wafer, respectively. N_{bulk} and N_{SERS} are the average number of molecules in the scattering area for non-SERS and SERS measurements. In this study, the number of probe molecules was estimated using Eq. 2 based on the assumption that the probed molecules were distributed uniformly on the substrates. N_A is the Avogadro constant; C is the molar concentration of the probe solution; V is the volume of a droplet; and S_{sub} is the area of the substrate.

$$\mathrm{EF} = \left(\frac{I_{SERS}}{I_{bulk}}\right)\left(\frac{N_{bulk}}{N_{SERS}}\right) \quad (1)$$

$$\mathrm{N} = \mathrm{CV}N_A S_{sub} \quad (2)$$

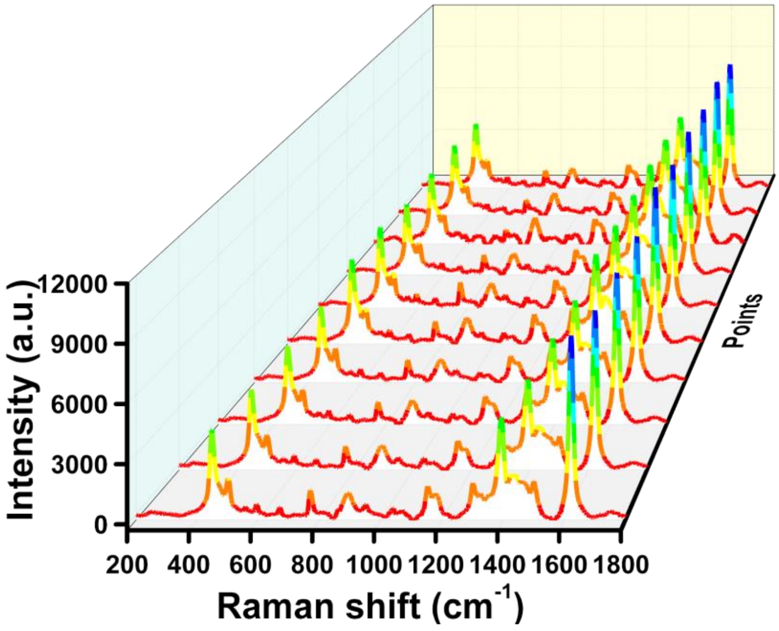

Fig. S4. SERS spectra of 2×10^{-5} M MB acquired on ten pieces of TiO_2 NTs.

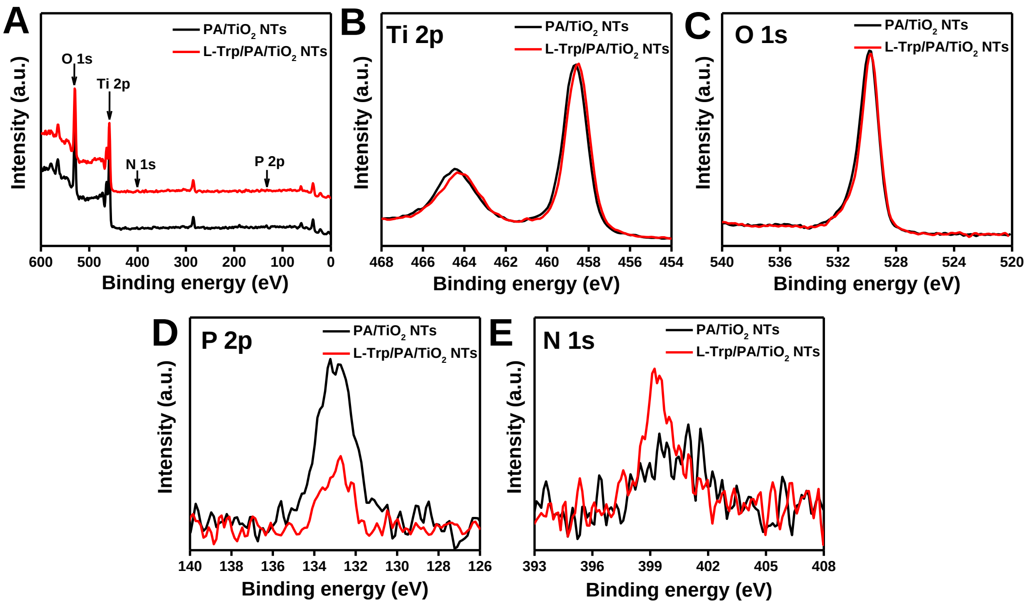

Fig. S5. (A) XPS survey spectra of PA/TiO_2 NTs before and after L-Trp modification. (B) Ti 2p signal, (C) O 1s signal, (D) P 2p signal, and (E) N 1s signal before and after L-Trp modified onto PA/TiO_2 NTs.

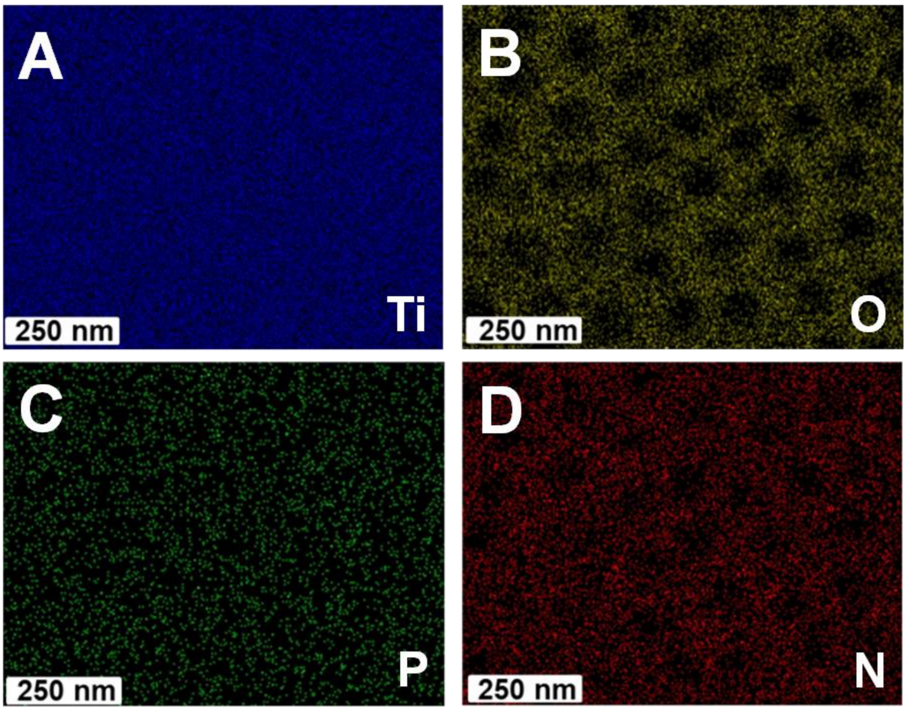

Fig. S6. The energy-dispersive X-ray spectroscopy (EDS) mapping images of L-Trp/PA/TiO_2 NTs.

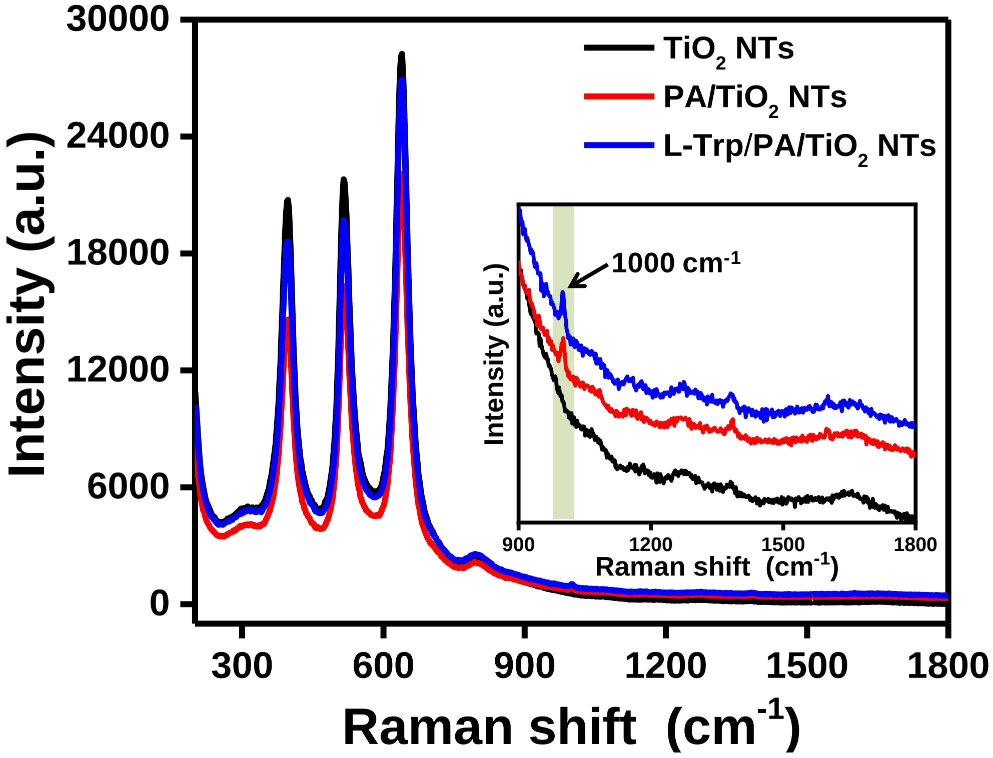

Fig. S7. Raman spectra of TiO_2 NTs, PA/TiO_2 NTs, and L-Trp/PA/TiO_2 NTs.

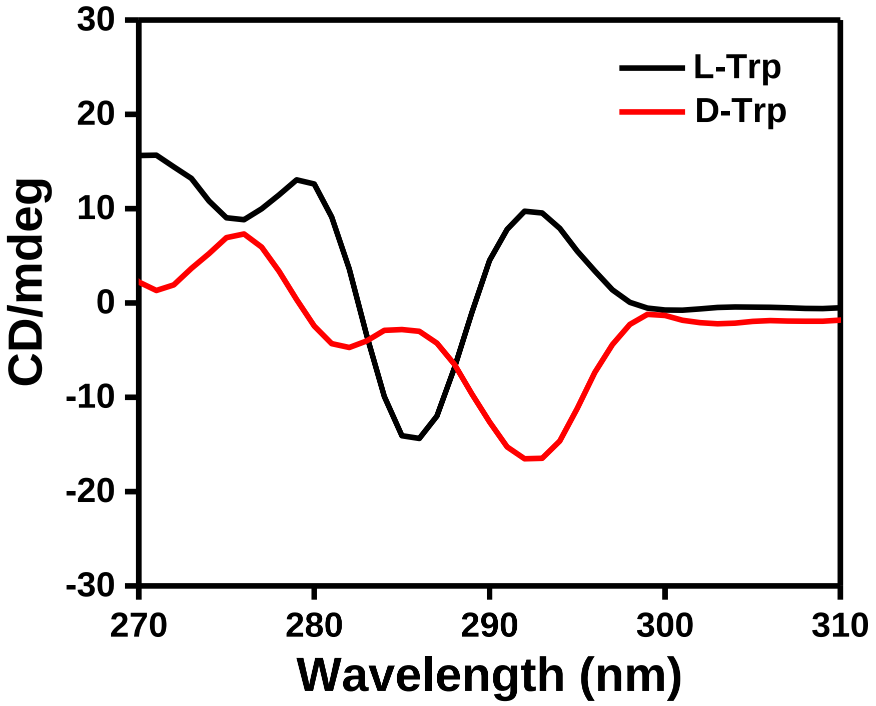

Fig. S8. CD spectra of L/D-Trp.

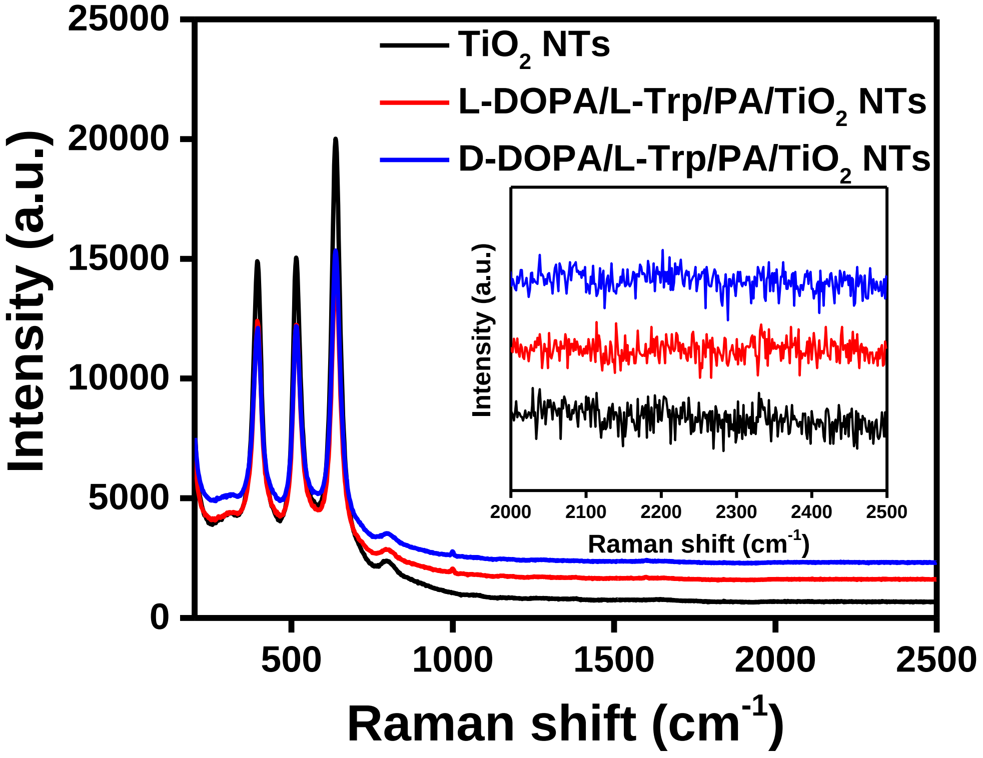

Fig. S9. Raman spectra of TiO_2 NTs, L-DOPA/L-Trp/PA/TiO_2 NTs, and D-DOPA/L-Trp/PA/TiO_2 NTs.

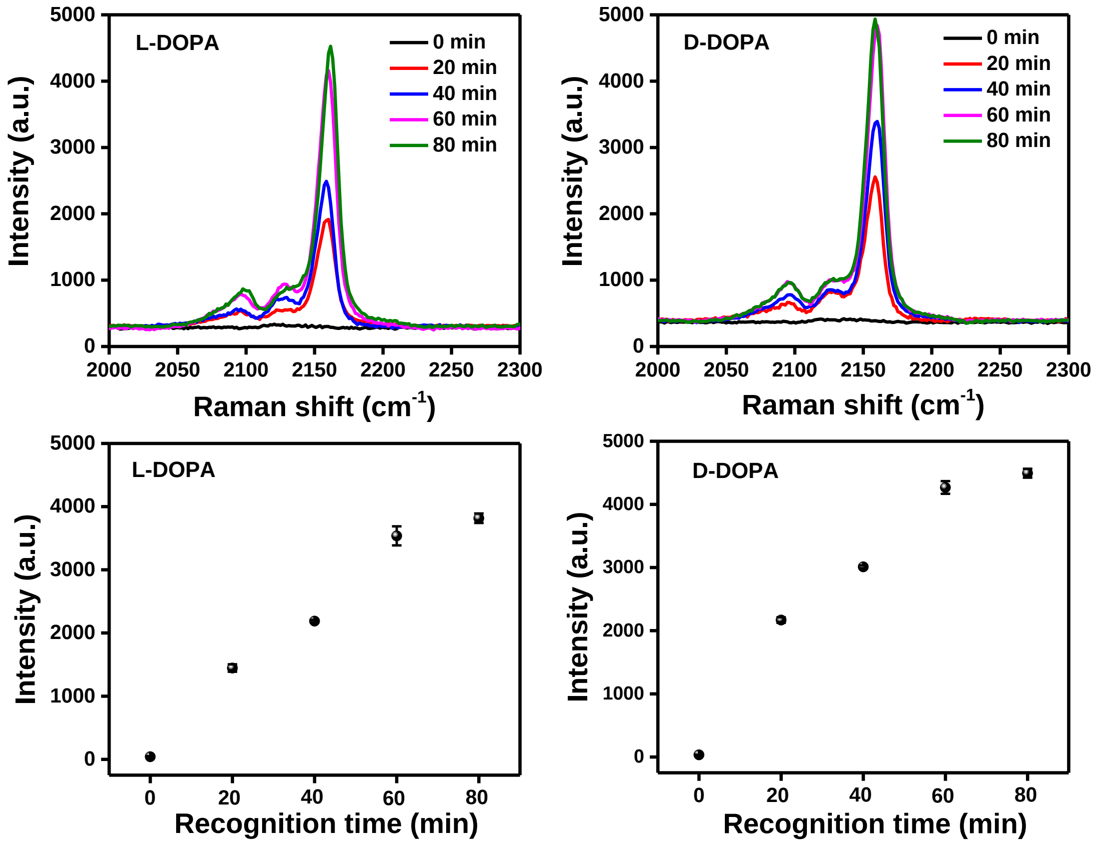

Fig. S10. SERS spectra for sensing (A) L-DOPA and (B) D-DOPA under different recognition time periods. The corresponding Raman intensity for sensing (C) L-DOPA and (D) D-DOPA.

As shown in Fig. S10, the Raman peak intensity at 2158 cm^{-1} increases with the chiral recognition time, and this trend slows down at 60 min.

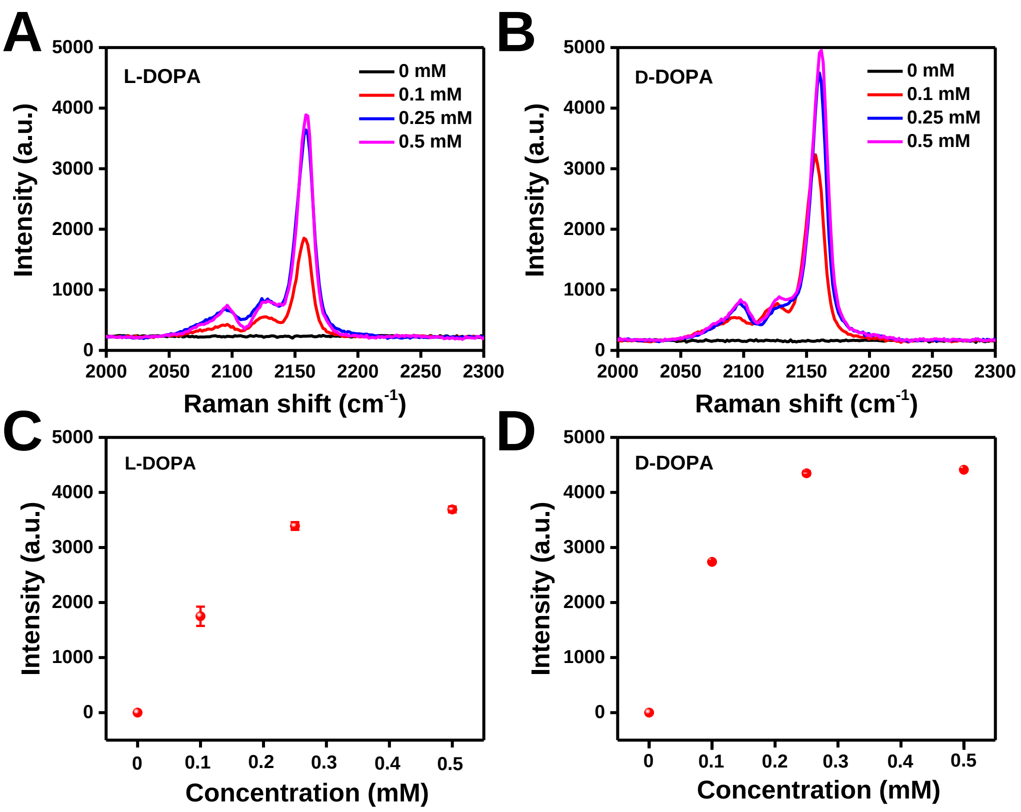

Fig. S11. SERS spectra for sensing (A) L-DOPA and (B) D-DOPA under different Fe^{3+} concentrations. The corresponding Raman intensity for sensing (C) L-DOPA and (D) D-DOPA.

It can be seen that the SERS signals of PB increased with Fe^{3+} concentration for L-DOPA and D-DOPA. The SERS signal intensity reached a plateau at a Fe^{3+} concentration of 0.25 mM for both cases. At this high concentration of Fe^{3+}, the reaction has nearly completed. Further increasing the concentration of Fe^{3+} does not shift the equilibrium too much (The intensity of the signal does not increase with higher concentration of Fe^{3+}). As shown in Fig. S11, the SERS substrate with D-DOPA consistently shows a larger PB signal than that of L-DOPA with any concentration of Fe^{3+}, suggesting more D-DOPA molecules were captured on the SERS substrate. This result was in agreement with the MD simulation.

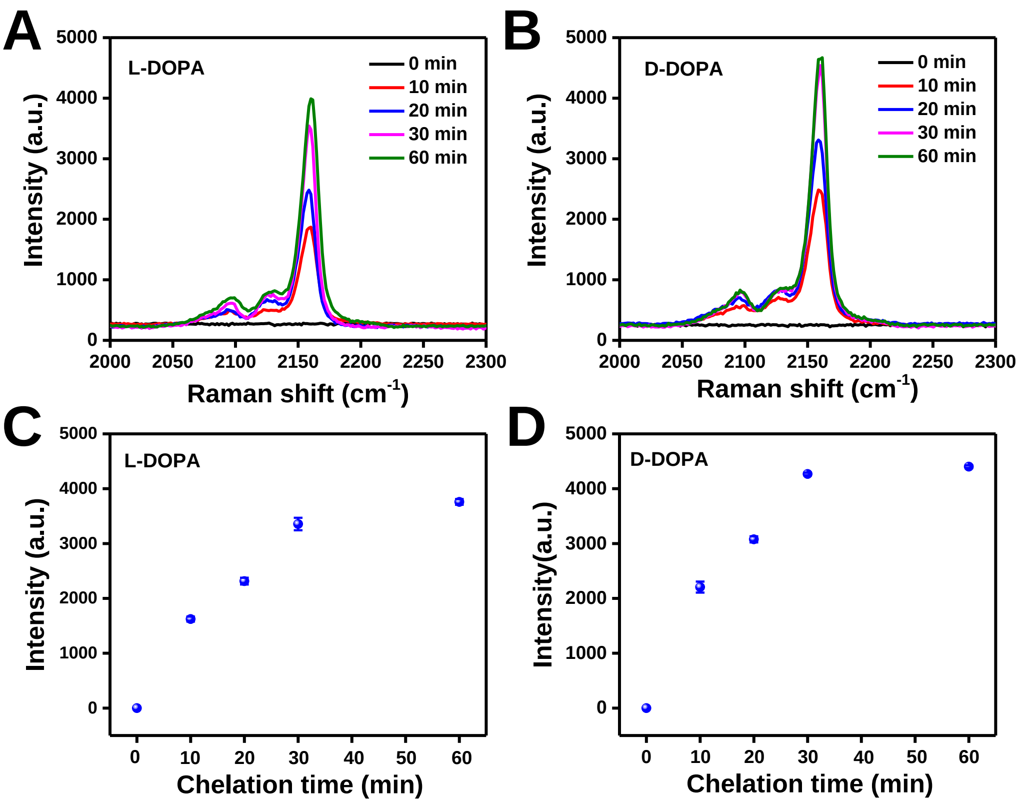

Fig. S12. SERS spectra for sensing (A) L-DOPA and (B) D-DOPA under different Fe^{3+} chelation time periods. The corresponding Raman intensity for sensing (C) L-DOPA and (D) D-DOPA.

As shown in Fig. S12, the changes in Raman peak intensity are also influenced by Fe^{3+} chelation time, and a stable value was achieved in 30 min.

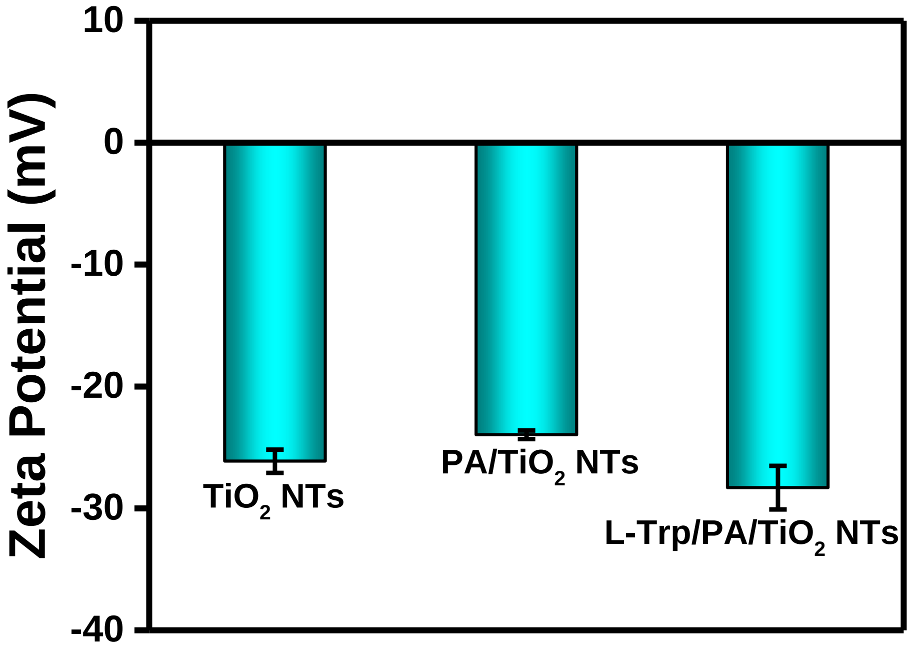

Fig. S13. Zeta potentials of TiO_2 NTs, PA/TiO_2 NTs, and L-Trp/PA/ TiO_2 NTs.

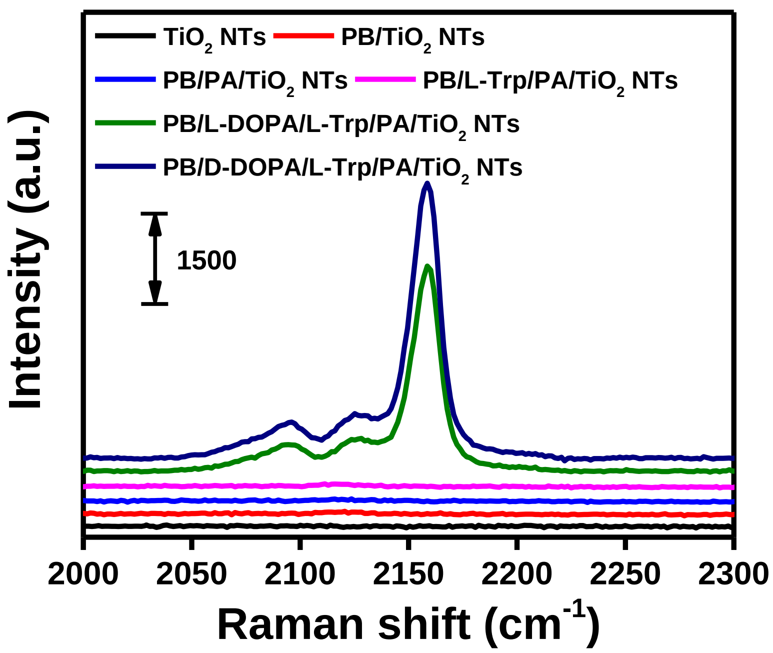

Fig. S14. SERS spectra of TiO_2 NTs, PB/TiO_2 NTs, PB/PA/TiO_2 NTs, PB/L-Trp/PA/TiO_2 NTs, PB/L-DOPA/L-Trp/PA/TiO_2 NTs, and PB/D-DOPA/L-Trp/PA/TiO_2 NTs.

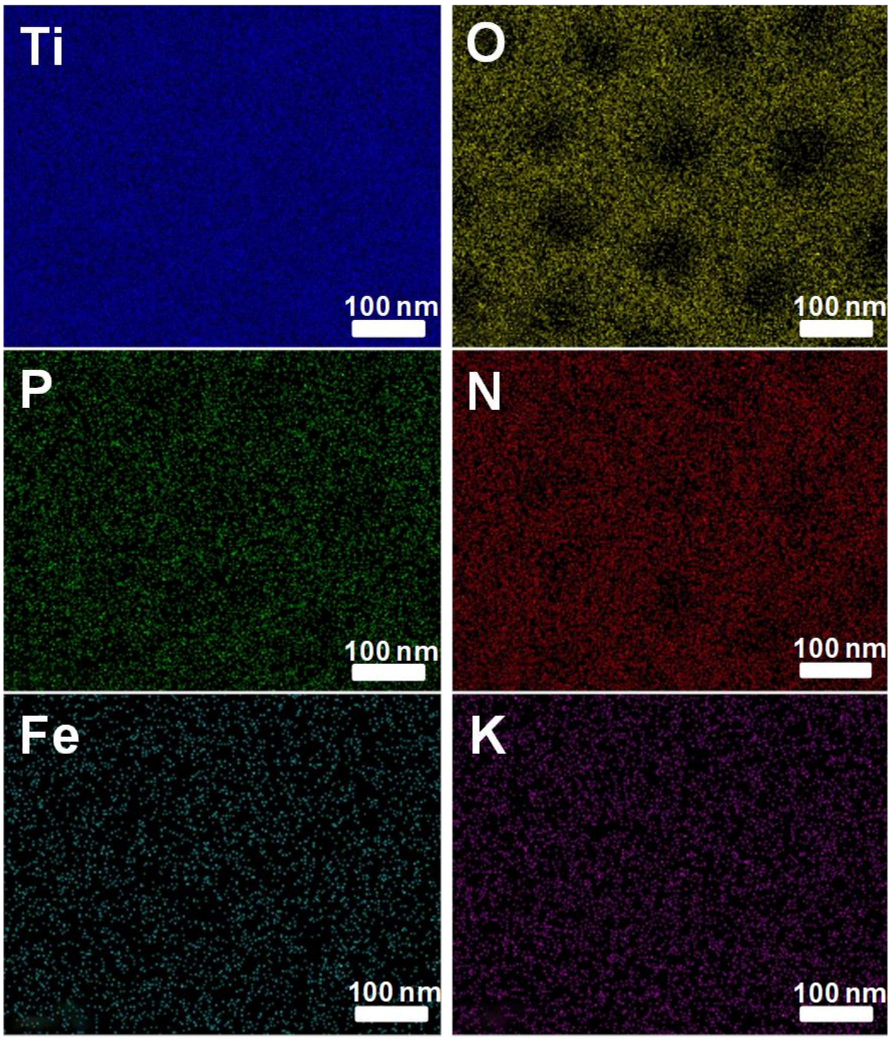

Fig. S15. EDS elemental mapping images of generated PB *via* DOPA recognition on L-Trp/PA/TiO_2 NTs.

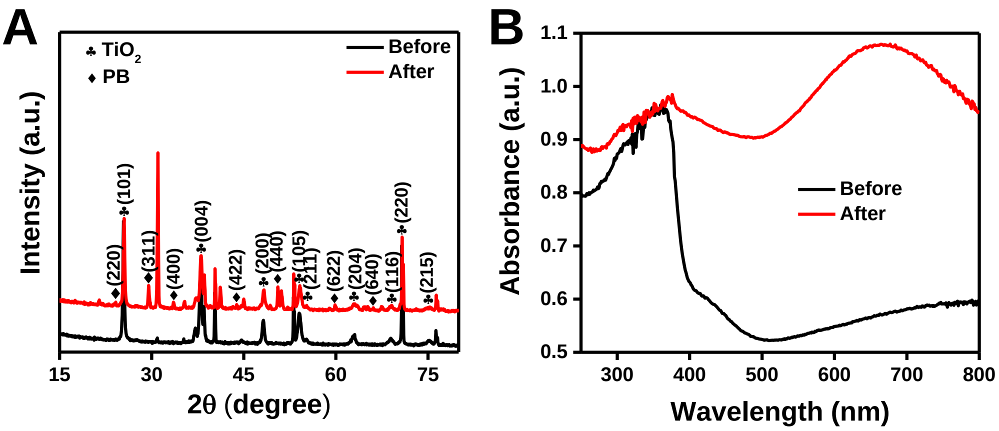

Fig. S16. (A) X-ray diffraction (XRD) patterns and (B) UV/Visible diffuse reflectance spectra of homochiral SERS substrate before and after PB generation.

The peaks appeared at 24.1 ° (220), 29.5 ° (311), 33.5 ° (400), 43.8 ° (422), 50.6 ° (440), 59.9 ° (622), and 66.1 ° (640), which can be indexed as the PB cubic space group *Fm3m* (JCPDS card no. 73–0687).[S2]

The UV-visible absorbance spectra verify the formation of PB *via* the broad absorbance band appearing from 480 nm to 800 nm, which can be attributed to the intermetal charge-transfer band from Fe(II) to Fe(III) in PB.[S3]

Table S1. Comparison of various methods for detection of L/D-DOPA.

Method	Mechanisms	Sensitivity data	Reference
L/D-cysteine modified gold nanoparticle based colorimetric assay	L/D-Cys as chiral selectors and Au NPs as artificial enzyme for enantioselective recognition of L/D-DOPA	1 μM	S4
L/D-tartaric acid grafted optical fiber probe based plasmon absorption bands shifts	Hydrogen bonding induced wavelength shift of plasmonic metal.	10 nM	S5
Gold nanodendrite modified glassy carbon electrode based differential pulse voltammetry	DOPA enantiomers were distinguished by a noticeable potential difference	0.25 μM	S6
Homochiral metal-organic frameworks based photoelectrochemical assay	the charge-carrier separation-induced photocurrent variation	0.24 μM	S7
Chiral CuO/CoO nanofibers based electrochemiluminescence method	CuO/CoO NFs as the catalytic activity center and chiral cysteine as the inducer of chiral recognition for enantioselective catalysis and recognition of L/D-DOPA	0.29 nM for L-DOPA; 0.31 nM for D-DOPA	S8
Signal amplification based SERS assay	Hydrogen-bonding interactions were amplified through the onsite growth of Prussian blue	0.13 pM for L-DOPA; 0.17 pM for D-DOPA	This work

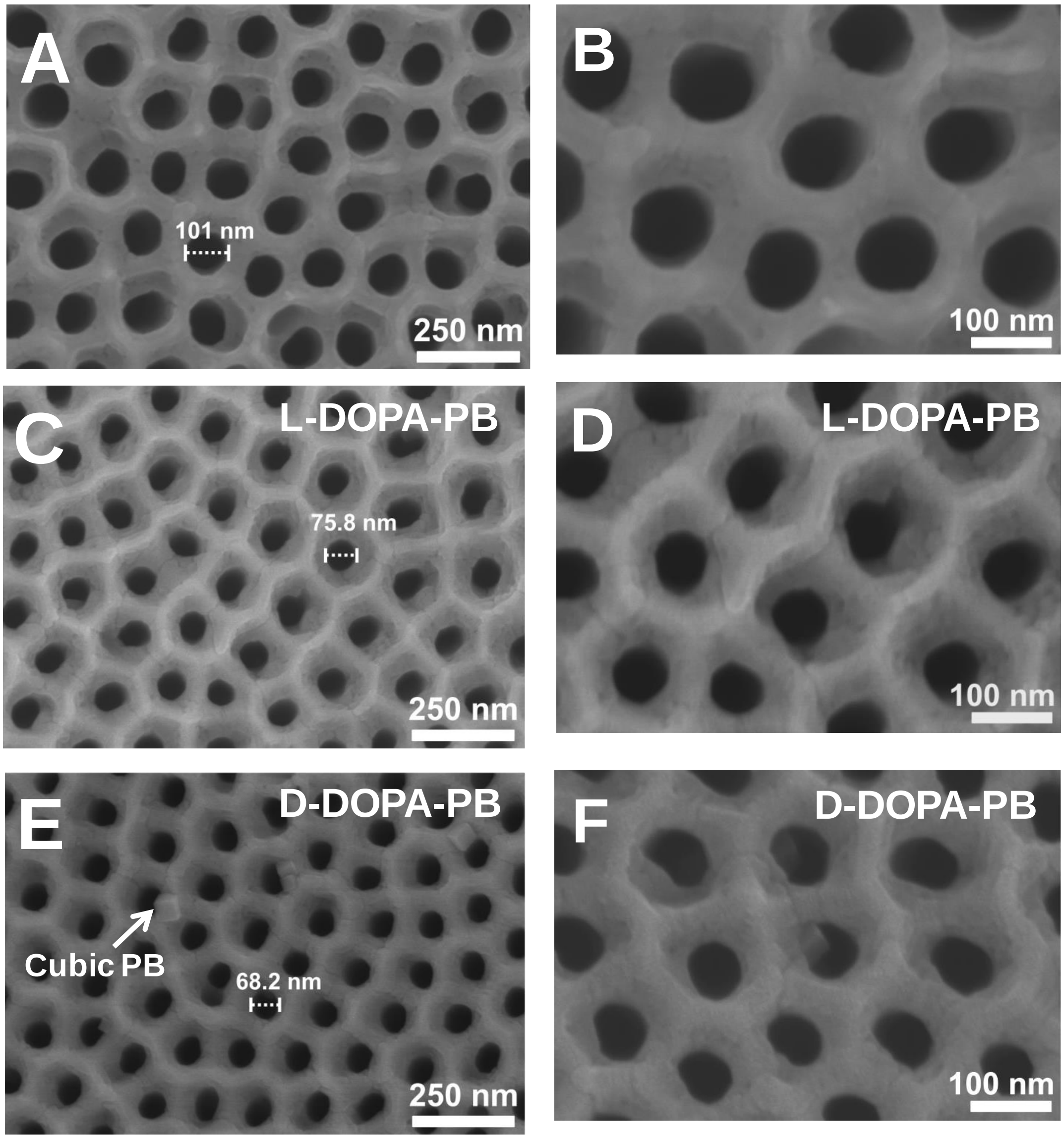

Fig. S17. SEM images of (A, B) bare substrate, (C, D) PB coated by L-DOPA recognition, and (E, F) PB coated by D-DOPA recognition.

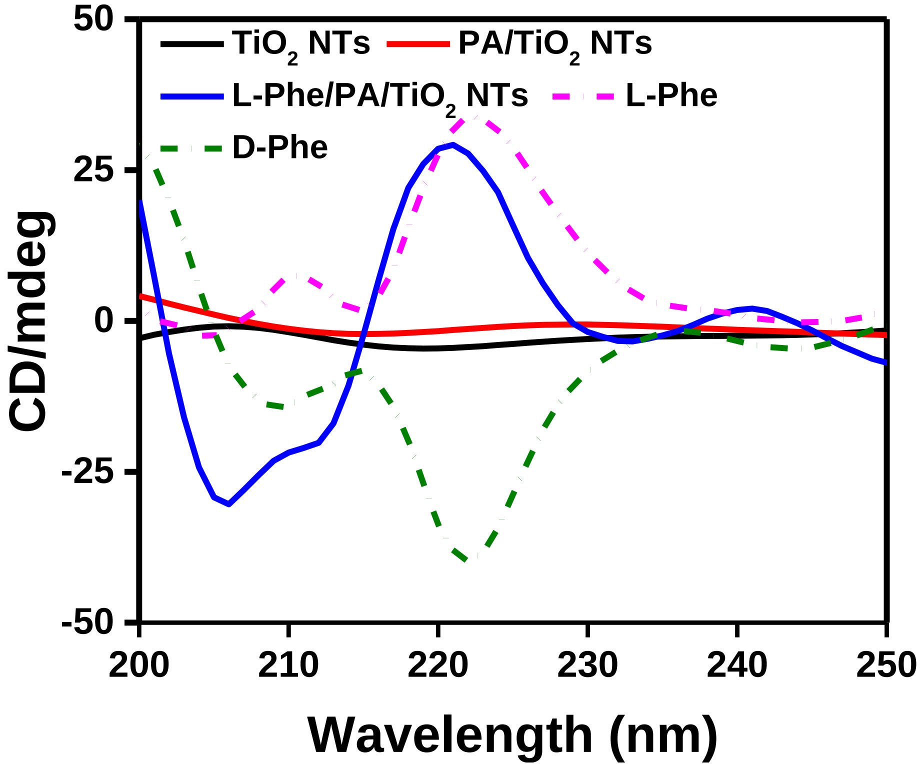

Fig. S18. CD spectra of TiO_2 NTs, PA/TiO_2 NTs, L-Phe/PA/TiO_2 NTs, and L/D-Phe.

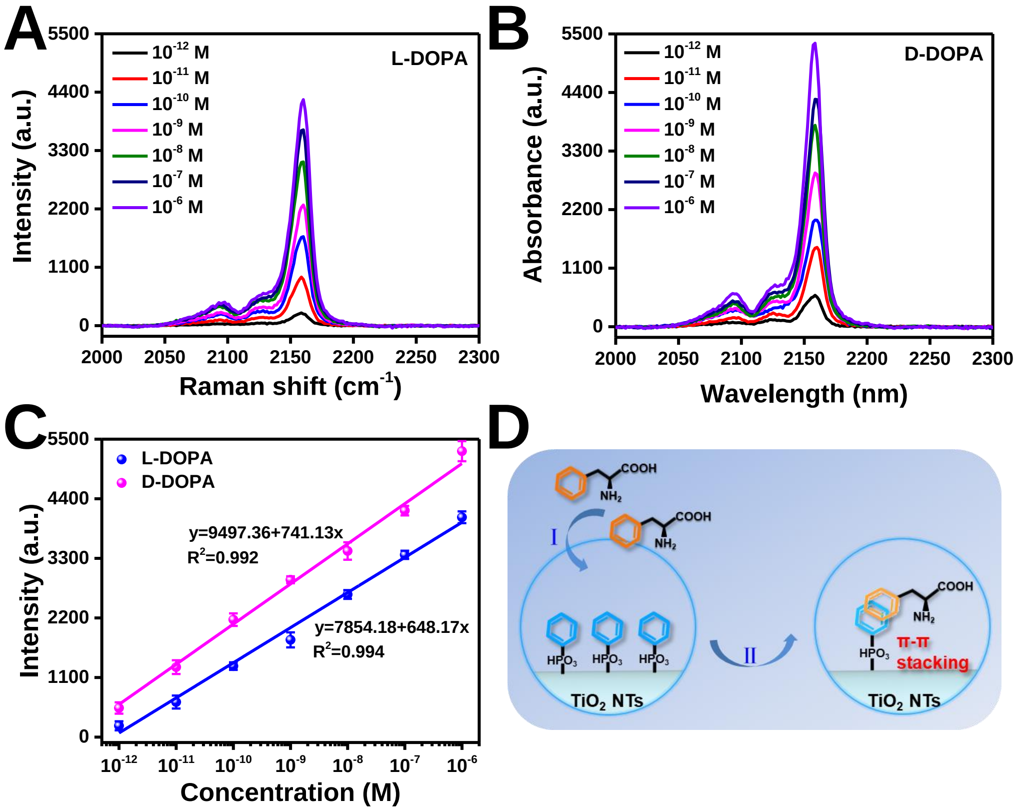

Fig. S19. SERS spectra of PB for sensing different concentrations of (A) L-DOPA and (B) D-DOPA on L-Phe/PA/TiO_2 NTs. (C) The corresponding Raman intensities at 2158 cm^{-1}. (D) Mechanism for anchoring L-Phe onto PA/TiO_2 NTs.

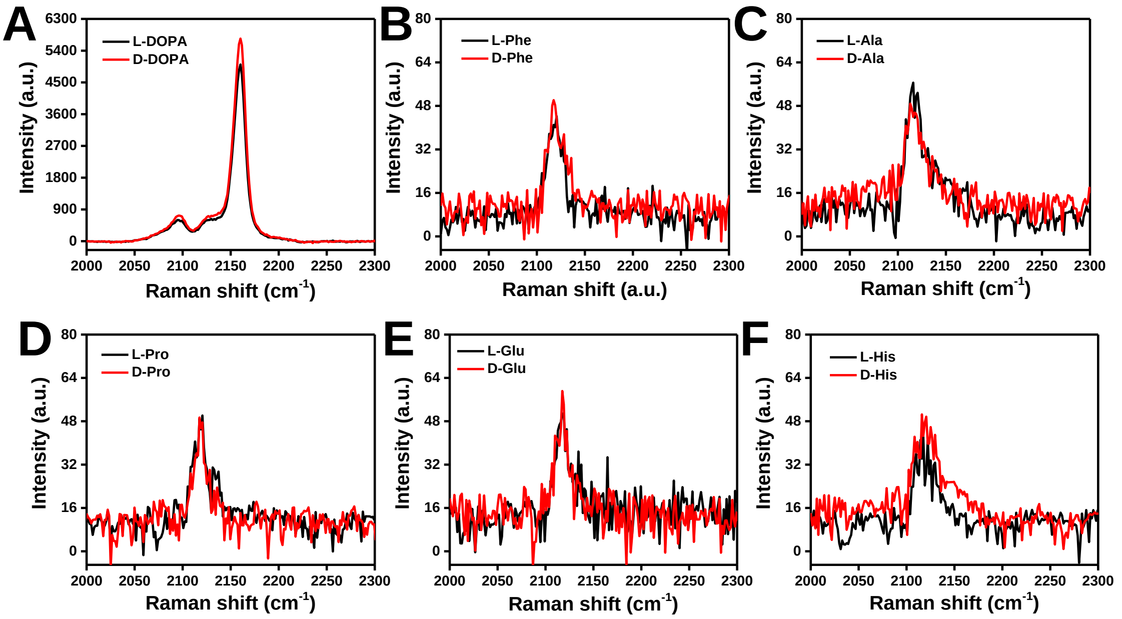

Fig. S20. SERS spectra of PB for sensing different enantiomers (A) L/D-DOPA, (B) L/D-Phe, (C) L/D-Ala, (D) L/D-Pro, (E) L/D-Glu, and (F) L/D-His.

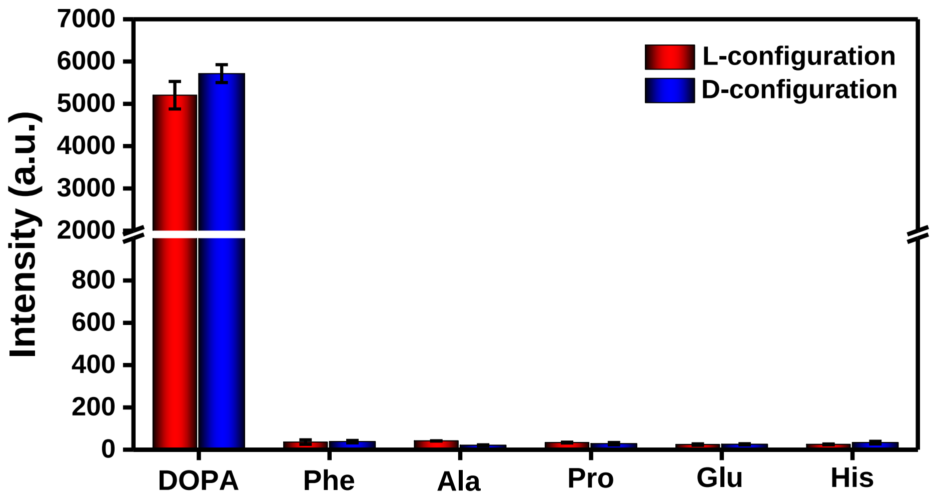

Fig. S21. Raman intensity at 2158 cm^{-1} towards different enantiomers. Error bars indicate the standard deviation of triplicate tests. The concentration of each enantiomer is 1 mM.

REFERENCES

(S1) J. Xu, H. He, X. Jian, K. Qu, J. Xu, C. Li, Z. Gao and Y.-Y. Song, *Anal. Chem.*, 2021, **93**, 9286–9295.

(S2) Z. Qin, B. Chen, Y. Mao, C. Shi, Y. Li, X. Huang, F. Yang and N. Gu, *ACS Appl. Mater. Interfaces*, 2020, **12**, 57382−57390.

(S3) Y.-Y. Song, W.-Z. Jia, Y. Li, X.-H. Xia, Q.-J. Wang, J.-W. Zhao and Y.-D. Yan, *Adv. Funct. Mater.*, 2007, **17**, 2808–2814.

(S4) Y. Zhou, H. Sun, H. Xu, S. Matysiak, J. Ren and X. Qu, *Angew. Chem. Int. Ed.*, 2018, **57**, 16791–16795.

(S5) E. Miliutina, O. Guselnikova, A. Kushnarenko, P. Bainova, P. Postnikov, V. Hnatowicz, V. Svorcik and O. Lyutakov, *ACS Sens.*, 2020, **5**, 50−56.

(S6) H. Lian, S. Huang, X. Wei, J. Guo, X. Sun and B. Liu, *Talanta,* 2020, **210** ,120654.

(S7) S. Zhou, J. Guo, Z. Dai, C. Liu, J. Zhao, Z. Gao and Y.-Y. Song, *Anal. Chem.*, 2021, **93**, 12067–12074.

(S8) Y. Song, S. Lu, J. Hai, K. Liang, S. Sun, G. Meng and B. Wang, *Anal. Chem.*, 2021, **93**, 11470−11478.
